# Maturing the design: challenges in maturing a first of a kind fusion power plant

**DOI:** 10.1098/rsta.2023.0412

**Published:** 2024-10-09

**Authors:** Debbie Kempton, Chris Waldon

**Affiliations:** ^1^ United Kingdom Atomic Energy Authority, Culham Campus, Abingdon, Oxfordshire OX14 3DB, UK

**Keywords:** maturity, capability, VUCA, STEP, MBSE, PLM

## Abstract

The design, delivery and operation of a large-scale infrastructure project are challenging at best. For the Spherical Tokamak for Energy Production (STEP) prototype powerplant (SPP), the challenges increased dramatically. In addition to being a large-scale infrastructure project, it is a cutting edge, first of a kind (FOAK) technology demonstrator. The design teams are working in a volatile, uncertain, complex and ambiguous environment, where technology is constantly emerging, maturing and changing. STEP will be unlike any power plant ever built and requires the development of new technologies and capabilities, but also a novel approach to planning and maturing the design. By taking a holistic view of the engineering life cycle from the start, the programme will be better positioned to achieve an SPP that is fit for purpose and can be used to show a path to ultimate commercial viability for subsequent power plants. This paper will review the key challenges in maturing a FOAK fusion power plant and look in depth at how the STEP team are maturing the required capabilities and planning to ensure successful delivery of the SPP.

This article is part of the theme issue ‘Delivering Fusion Energy – The Spherical Tokamak for Energy Production (STEP)’.

## 1. Introduction and context

To mature the design, it is necessary to consider the challenges and opportunities required for successful delivery to set the context for the identification and determination of maturity and planning enablers. As several of the papers in this special edition speak in detail to design and programme challenges [1,2], only a short summary of the programme’s key characteristics and required capability enablers is provided to set the context for the wider capability maturity and planning approach. The key defining characteristics of STEP can be summarized as follows:

— *STEP is a mega project*: Significant in both budget (£Bs) and time (multi-decade), delivery of the SPP will involve multiple partners, organizations, suppliers and regulatory bodies, as well as a myriad of government stakeholders and agencies at the local and national levels. The oil and gas industry, aviation and transportation industries have provided useful context [3–6], with the Nautilus nuclear submarine programme [7] and the Space Shuttle programme [8] being especially edifying with respect to qualification and integration approaches.
— *STEP has significant interdependencies*: As a large, complex and complicated system of systems, there is often a need to compromise to achieve the best for the overall plant.
— *STEP is first of a kind* (*FOAK*): The SPP is a broad portfolio of nascent technologies, developing concurrently with emergent properties in an interrelated system over a 40 year horizon. The design of today will evolve considerably through its operational life.
— *Industry is inexperienced with respect to fusion*: As there is a lack of experience in wider industry with fusion, in general, and fusion power plants, in particular, the programme must allow, and plan for, enough time and funding to upskill the supply chain while leveraging their extant capabilities in complex engineering design and manufacturing.
— *Fusion regulation is a nascent space*: While the regulatory agencies responsible have been identified and legislation signed into law [9], the practical application of fusion regulation is untested and there is a danger of unconsciously falling back on fission norms which may over-constrain the design space given the different nature of hazards [10].
— *STEP requires a digital twin*: Including not just modelling and simulation, but all computer-aided design (CAD) models, decisions, design options, trades, requirements and cost and performance data, the digital twin will both enable the next organization to build future commercial fusion plants and allow the STEP Programme to optimize the SPP through life [11,12].
— *Cost considerations are equally important as design considerations*: It is not enough to mature a design that meets functional and performance requirements, the SPP must also demonstrate a realistic route to commercialization [13].

When looking holistically across these defining characteristics, several themes emerge: the need for collaboration and robust information management, the need for model based systems engineering (MBSE), a ‘best for programme’ mindset and the ability to work in an uncertain environment. While addressing these is necessary, it is not sufficient as key engineering capability enablers must also mature to create the desired outcome. These enablers can be summarized as follows:

— *People and culture*: In addition to upskilling for new tools and techniques, the commercialization of fusion requires staff to move from a research regime to a delivery regime, necessitating a change in both culture and approach [14].
— *Process, tools and infrastructure*: While the STEP programme has already begun implementing MBSE capability and a product lifecycle management (PLM) system, both must be developed and matured at pace with the needs of the programme and desired maturity targets. In addition, STEP will require significant modelling and simulation efforts as key requirements must be validated *in silico*, which requires high-performance computing (HPC) coupled with a robust IT infrastructure and back office [15].
— *Data and information*: Given a key objective of the STEP programme is technology demonstration, the programme will need to generate appropriate data to demonstrate that the technology development activities are delivering the right outcomes at the right time, while simultaneously minimizing overall risk and maintaining design progress. Maturity metrics must look across the maturity of the design and its fitness for purpose (‘goodness’), account for risk, cost and availability, and also align with existing industrial programme and regulatory measures (e.g. the Royal Institute of British Architects (RIBA) stages, Building Information Modelling (BIM) or Health and Safety regulation) to ensure that the programme can communicate and manage effectively across the wide range of partners and stakeholders while concurrently creating a useful (and usable) digital twin.

## 2. The path to maturity

As a FOAK, STEP will be unlike any other power plant ever built and will require the development of new capabilities to see it successfully delivered. Pushing physics to the edge of what is currently known, betting on what will be available in the future and continuing to research new and exciting possibilities all find their place in maturing the design approach. And while there are indeed similarities to fission plants, coal-fired plants, wind farms and solar arrays, STEP will in several ways need to blaze a new trail. Furthermore, it is crucial that the entirety of the product life cycle, from concept to disposal, is considered both in the instantiation of the design and also in the capabilities required to enable that design. The programme will not only require cultural changes but will also need to adopt new ways of working, support the development of new regulatory practices, develop new technologies and create information out of big data at both the management and delivery levels. Therefore, some of the key considerations in maturing the design are understanding relationships in a system of systems, identifying design drivers, defining design sequencing, understanding and managing maturity and building design confidence. Each of these considerations is discussed in subsequent sections.

### (a) Understanding relationships in a system of systems

**Figure 1.**
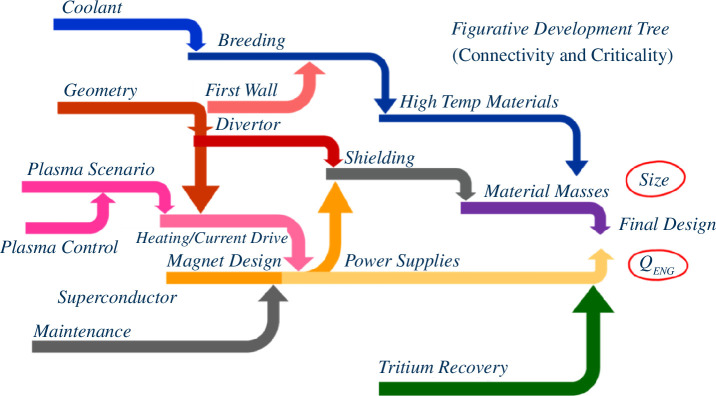
The SPP interconnected design challenge.

As discussed previously, the SPP is a highly complex and interrelated system of systems. However, its delivery will also be paced by the emergence of the design specification. In other words, as a FOAK, there is no extant specification and requirements will evolve and change as the design evolves. And while STEP will certainly learn lessons from ITER, DEMO, CFEDR and other ongoing fusion initiatives, the design of compact fusion reactors critically depends on advancing technology and navigating proximity to technological limits and tipping points. This technological sensitivity is heightened for compact devices and is intrinsic to stabilizing their design [2]. Figure 1 conceptually illustrates this challenge and shows how changes in one SPP system (or a set of requirements) can ripple through multiple systems.

As a result of this interconnectivity, the STEP design space has extensive uncertainties in the delivery pathway worsened by many moving parts, causality breakdown and the multiple significant decisions to be made. There are many strongly interacting elements (tensions) both within and between the physics, technology, engineering and capability enablers, all of which make the pathway appear opaque. In addition, and unusually for a major infrastructure programme, the integrated design is built on a nascent technology development portfolio that must continuously deliver increasing levels of confidence that functional demands can be met within the constrained space. This is analogous to the Space Shuttle programme [8] where in Phase C, two years immediately after the contract award, there were many changes to the design as challenges were better understood through analysis and experimentation. The baseline went through six configuration changes in an ‘architectural-system refinement’ design phase with the addition, deletion and reinstatement of various systems, subsystems and component parts.

**Figure 2.**
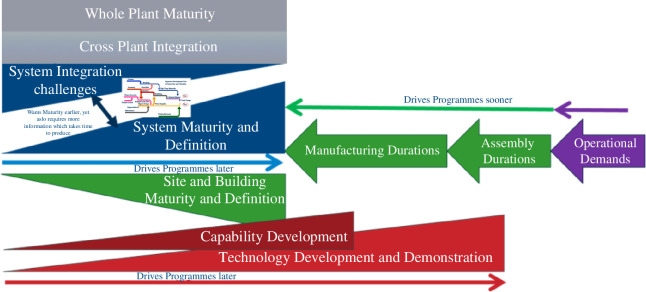
Design tensions in a first of a kind, emergent development programme.

Therefore, owing to this extreme degree of interconnectivity, a highly contested critical path that is influenced by chronology, connection and sensitivity emerges. However, understanding the critical path, while necessary, is not sufficient to determine design sequencing. The fundamental tensions in the programme must also be addressed—the desire to ‘move right’ owing to volatility, uncertainty or ambiguity in key systems of nascent technologies must be balanced against the need to ‘move left’ to facilitate the understanding (and mitigate the impact) of key integration challenges, long lead procurement, supply chain development and the development of whole plant requirements. Figure 2 illustrates these tensions which influence not only chronology but also risk.

### (b) Identifying design drivers

**Figure 3.**
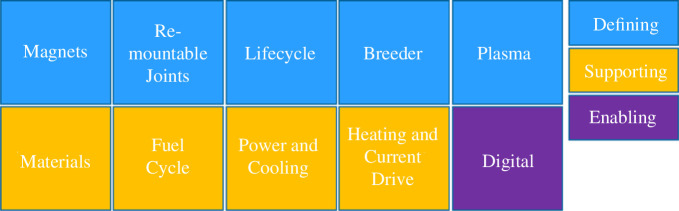
Defining, supporting and enabling systems.

By jointly considering programme tensions and interconnectivity a view of defining and supporting systems has emerged. Defining solutions are those that compel the design to take a particular direction by shaping key design or performance requirements and system architecture while supporting solutions are those that enable whole plant instantiation and are subsequently derived from the defining solutions. For the SPP, the space available for the radial build, the surface area available for handling heat, particle flux and tritium breeding and the architecture and robotics strategy necessary for rapid assembly and maintenance have dictated which are the defining solutions and, subsequently, the supporting solutions emerged as a result. Additionally, there is a ‘hidden’ enabler which must be considered as it is fundamental to the successful delivery of the programme, which is the digital twin. While not a physical deliverable, it is a key capability enabler for *in silico* qualification (i.e. reduced physical testing), data exchange and plant optimization and its maturity has been considered and developed alongside the defining and supporting solutions. As a point of reference, one of the significant lessons learned from the International Thermonuclear Experimental Reactor (ITER) was the amount of time and effort required to retrofit a PLM system and rework systems and data to create a digital twin. STEP has started as it means to go on, to realize all the benefits of a digital twin throughout the engineering and product life cycles. Figure 3 illustrates this leader/follower concept of defining and supporting solutions and identifies how it has been applied to the SPP.

Magnets, re-mountable joints (RMJ), the breeder and plasma systems and the plant life cycle (e.g. through life maintenance) are the defining solutions for the SPP. The resulting supporting solutions are subsequently derived, which allows STEP to structure the delivery of the programme in such a way as to build confidence in the less certain and more ambiguous systems first. Note that materials are considered a supporting solution as multiple considerations, especially breeder design choices, have informed material choices, rather than materials dictating the design. This then means that material optimization programmes may need to be separately undertaken to deliver the FOAK SPP.

### (c) Defining design sequencing

**Figure 4.**
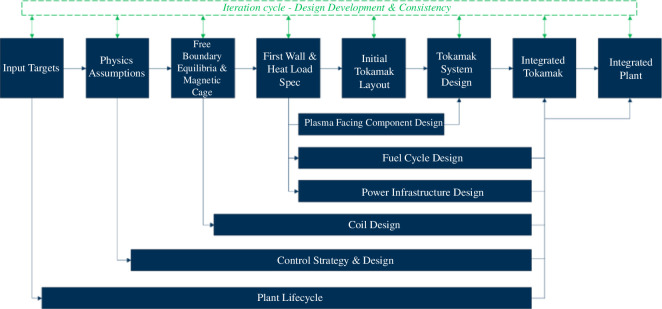
SPP concept design sequencing.

Once design interdependencies, tensions and defining and supporting systems were understood, a plant design sequence was developed. Figure 4 illustrates a simplified version of the concept design sequencing for the SPP and provides an order of operations to allow the design to be understood and planned. However, it is worth noting that while it appears very linear, there are multiple feedback loops and often partway through the design a pivot or modification in direction was required. The design sequence is a touchstone that allows the programme to understand when additional systems and subsystems must be revisited, but also aids in the understanding of risk. Notably, even the conventional tokamak development pathway is not without risk, as demonstrated by the European DEMO G1 review [2]. Public nuclear fusion programmes typically follow a linear innovation model, while disruptive fusion start-ups pursue agile innovation [16]. For instance, the Chinese fusion engineering DEMO reactor (CFEDR) informed the construction of the Comprehensive Research Facility for Fusion Technology (CRAFT). Initial development activities in CRAFT focused on the superconducting magnet research system and the divertor research system. In the coming years, additional R&D efforts will validate key technologies across all subsystems, particularly in plasma-facing materials, high-temperature superconducting materials and large heating power systems [17]. Hence, design sequencing is an essential tool for specifying required maturity, facilitating understanding as to how far a system or subsystem can be allowed to progress ahead of others before risking nugatory work, and understanding where collaboration may be desirable.

**Figure 5.**
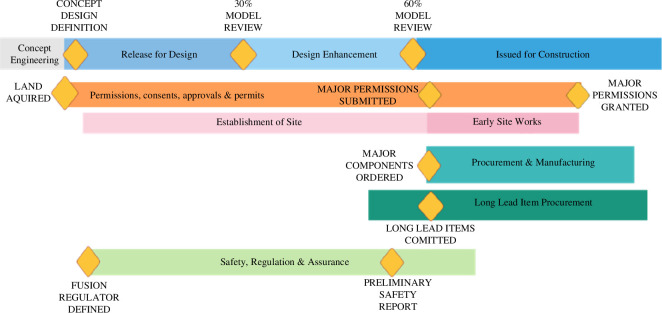
Critical interdependencies.

In terms of the wider programme schedule, figure 5 shows a notional programme schedule which couples the engineering activities with the site and other regulatory concerns.

### (d) Understanding and managing maturity

Finally, in order to mature the design, a balance must be struck between design maturity and risk appetite. To achieve this balance, a clear identification of both factors has been considered. STEP has a unique challenge in terms of maturing technologies in parallel with maturing the design. As a FOAK, traditional views of maturity will not fully describe the SPP, nor will risk be reduced by taking a narrow view of performance against requirements or slavishly following proscribed standards. Even commonly used measures like technology readiness levels (TRLs) or the National Aeronautics and Space Administration (NASA) concept maturity levels (CMLs), which the programme has used to date are challenging as it is not possible to provide an equivalent environment for testing without STEP itself, leaving many key technologies unable to progress beyond TRL4 [18] or CML5 [19]. Therefore, going forward, when defining maturity levels for STEP, maturity of methodology, maturity of design and maturity of understanding are now being considered.

Maturity of methodology concerns the ability of the programme to execute the engineering (i.e. how it is done). As a FOAK, traditional engineering approaches must be refined and redefined in a way that allows them to evolve as understanding develops. This is similar to the Nautilus nuclear submarine programme which was developed in the 1950s [7]. In both cases, the proposed operating space is a large extrapolation from current empirical experience and a full-scale integrated precursor testing is not feasible almost by definition. Nautilus took an empirical approach even though there often remained many unanswered questions and there was not always assurance that a satisfactory solution would ever be found. One could argue that the same is true of a compact reduced cost tokamak. Therefore, the programme is currently refining and developing ways of working, especially embedding the use of MBSE, digital twins and *in silico* qualification. Methodologies are being considered to not only capture the design but also allow the programme to optimize and predict performance.

**Figure 6.**
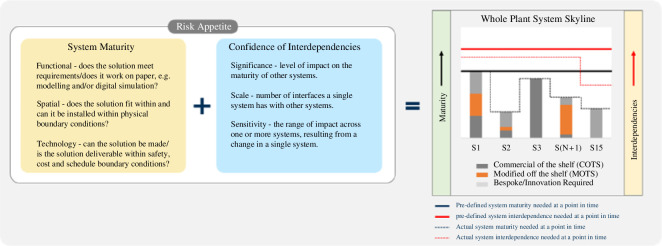
System skyline.

Maturity of design considers the ability of the SPP design to not only meet requirements but also to have sufficient margin (i.e. is the design deliverable). To illustrate the requisite level of maturity needed within (and across) one or more systems at a single point in time a series of system skylines have been defined at pre-determined times during Tranche 2a. The skylines, shown in figure 6, combine aspects of system maturity with confidence in interdependencies between systems and help inform risk exposure and risk mitigations. The position and shape of the continuous black (maturity) and red (interdependencies) lines create the skyline. At any given point in time, the skyline will be determined on a need and risk basis, i.e. a level of readiness needed to enable the programme to proceed with significant decisions. The gap between the target and actual skylines will inform areas where effort is needed to acquire design coherence and/or mitigate risk.

Finally, maturity of understanding interrogates outcomes (i.e. is the design stable). It is about reviewing the holistic behaviour of the plant as well as the processes, tools and results to characterize the degree of uncertainty attendant in those results. As discussed previously, the large degree of interdependency and the emergent nature of the technology require understanding to develop at pace with the system level design. However, gathering knowledge and building understanding may require revisiting entire methodologies or designs. Therefore, a critical path to successful design is a refinement of understanding. Maturity of understanding as a consciously interrogated maturity viewpoint allows the programme to continuously sense check approaches and results for fidelity and stability.

**Figure 7.**
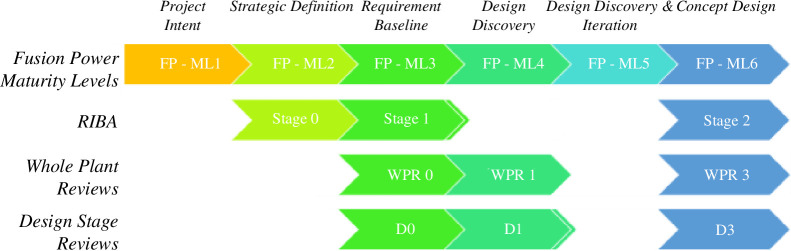
Alignment of whole plant maturity targets.

In terms of maturity management, identifying, understanding and mastering interdependencies between systems will require design iterations before an optimal balance within and between systems is achieved. A common level of whole plant maturity (including buildings and infrastructure) will not be attained at a single point in time. Different systems will mature at different rates and the level of confidence in the interdependencies within and between systems will be iterative and vary at any single point in time. This reinforces the need for iterative, incremental capability development as previously discussed and shown in figure 7. Fusion power maturity levels (FP-MLs) have been assessed for each system identified within the plant breakdown structure (PBS) which enables the maturity targets to be set against specific design gates—whole plant reviews (WPR). The targeted maturity levels across the PBS will reflect the importance and/or technical risk of a particular system. All individual systems will be expected to progress through a series of design reviews, and the FP-ML provides clear guidance to the expectations of each review stage. The maturity levels have further been aligned with the Royal Institute of British Architects (RIBA) stages to ensure that the tokamak and plant design interdependencies are considered. Figure 7 is an illustrative example of how maturity is considered across the whole plant.

### (e) Building design confidence

Once the design drivers and sequencing were understood, confidence in the design planning and execution needed to be built. As STEP is a multi-year endeavour, confidence must incrementally increase, so the programme can satisfy itself that the design is progressing but also generate (and demonstrate) value for money. This has been approached by the programme in three ways: delivering incremental capability, demonstrating technology and realizing digital enablement.

#### (i) Delivering incremental capability

**Figure 8.**
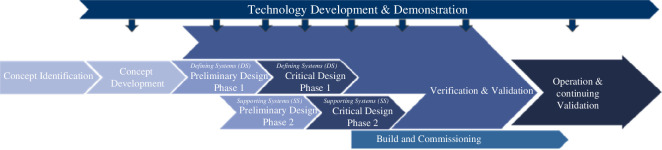
Iterative, incremental capability development to build design confidence.

Post the conceptual design phase, when all systems have been brought to a common maturity level, the implementation of the defining systems (the leader) and supporting systems (followers) will be enacted. Figure 8 illustrates this concept of staggered (or spiral) development, including incremental technology development drops and incremental verification and validation (physical and *in silico*), which allows confidence to be built incrementally over time.

To achieve this, design and technology streams will run in parallel, with each planned around the success of the other. Milestones and gate reviews will be used to maintain alignment and adjustments will be made to incorporate favourable and unfavourable results in both directions.

Finally, a description and definition of the maturity for each system at pre-determined (pivot) points will be developed ahead of major programme commitments. These will provide interim target positions to focus efforts and help map the timing (and pace) of design development. During preliminary design, design development will have proportionally greater focus on addressing performance matters (functional and spatial requirements), to produce an integrated whole-plant solution, of adequate coherence.

Coherence between the concurrent plant and building design will be scrutinized and ensured through integration layer design reviews that orchestrate facility and plant system layouts as an integrated product with all differing maturities therein [20]. Particular consideration is given to the highly nuanced combinatory requirements of high-performance scientific technology, high-hazard plant and active facility design.

#### (ii) Demonstrating technology

A large part of building design confidence is regular and increasing demonstration of key technologies and processes [16]. The imperative for the design lies in its responsiveness to feedback derived from technology prototyping and testing, ensuring a continuous process of refinement. The developmental trajectory is intricate and characterized by numerous decision points. Recognizing the infeasibility of exploring every avenue, premature pruning is cautioned against owing to its potential detrimental effects. Therefore, in adherence to the lessons learnt from analogous endeavours, execution of a meticulous failure modes and effects analysis (FMEA) for innovative concepts is not only desirable but fundamental. This analysis, distinct from risk assessments, discerns potential failures in various contexts—full, partial or complete. FMEA thus illuminates critical escape routes in anticipation of potential failures. The overarching strategy advocates regular assessments, adaptability, inclusive feedback, proactive recovery planning and the unequivocal readiness to discard when the journey appears to have reached its conclusion.

**Figure 9.**
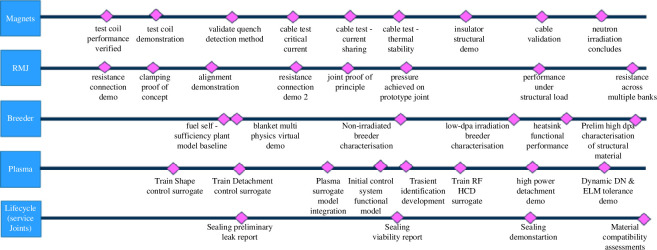
Indicative development of defining systems.

As stated previously, technological priority has been placed on the defining systems, i.e. those core to the compact concept: precise control of the plasma, reduced radial build, thermal management and breeding within space constraints. Deeper within these thematic headings lie device-defining parameters and configurations that have been encapsulated in verification and validation plans. Figure 9 illustrates an indicative technology demonstration roadmap for the defining systems, which has been sequenced to take advantage of the FMEA approach discussed and thereby build confidence.

#### (iii) Digital enablement

As the design and technology must mature simultaneously, successful delivery requires digital enablement [21]. Given the intricacies of the design, an MBSE core has been implemented to ensure self-consistency and efficacy within the PLM system [22,23]. MBSE has been shown to provide considerable benefit over the upfront investment as shorter communication pathways are key as the programme exponentially grows [11,24]. The PLM system provides a common reference, and this shared context facilitates efficient communication as integrated delivery team (IDT) members can anticipate each other’s needs and concerns with real-time updates, document sharing and virtual collaboration. Communication flows more directly and decisions can be made promptly, allowing for agile responses to unforeseen issues or design changes. Fewer intermediaries mean less time lost in relaying information and quick feedback loops enhance problem-solving and prevent bottlenecks. Most importantly, MBSE and the PLM system together provide a single source of truth and build confidence in both the design and the design process.

Finally, it is worth noting that neither tools nor infrastructure need to be fully implemented on day one of the programme. Just like the design process, a phased delivery, rather than a big bang, allows the capability to mature as the programme matures. This ensures focus on the right skills, tools and capabilities at the right time to support the phased delivery of the wider programme.

## 3. Conclusions

In maturing the SPP, there is no prescriptive or ‘right way’ through it—it is an evolving and emerging undertaking which requires a holistic view of both the programme and product life cycle to create a balanced outcome. Capabilities and plans must mature and evolve within guiderails, not rigid lines of development. However, while there are significant challenges in creating, maturing and delivering the SPP in an emergent space (both technologically and regulatory), these are not new challenges. For STEP, this phase of the programme focuses on limited but targeted physical demonstrations, the results of which will inform our modelling and simulation efforts, thereby building confidence in the design and the digital twin, allowing the design to mature as a FOAK prototype power plant. Successful engineering in STEP requires concurrent development of knowledge and design, driven by a clear focus on increasing maturity and enabled by an iterative approach that caters for inherent uncertainty. Existing engineering and science tools, techniques and approaches while informative are not sufficient to mature a FOAK prototype power plant. Management judgement will also be fundamental to maturing STEP and enabling the engineering in such a way as to allow the programme to flourish.

## Data Availability

This article has no additional data.
